# Preoperative Predictors of Late Aortic Expansion in Acute Type B Aortic Dissection Treated with TEVAR

**DOI:** 10.3390/jcm12082826

**Published:** 2023-04-12

**Authors:** Zhiqiang Dong, He Yang, Gang Li, Xinyang Xu, Hong Liu, Jiaxi Gu, Minghui Li, Weidong Gu, Yongfeng Shao, Buqing Ni

**Affiliations:** 1 Department of Cardiovascular Surgery, The First Affiliated Hospital of Nanjing Medical University, Nanjing 210003, China; 2 Department of Thoracic Surgery, The First Affiliated Hospital of Nanjing Medical University, Nanjing 210003, China

**Keywords:** aortic expansion, predictor, type B aortic dissection, aortic repair, aortic remodeling, adverse events

## Abstract

Background: A patent false lumen (FL) in patients with thoracic endovascular aortic repair (TEVAR)-treated type B aortic dissection (TBAD) can cause a significant risk for late aortic expansion (LAE). We hypothesize that preoperative features can predict the occurrence of LAE. Methods: Sufficient preoperative and postoperative follow-up clinical and imaging feature data for patients treated with TEVAR in the First Affiliated Hospital of Nanjing Medical University from January 2018 to December 2020 were collected. A univariate analysis and multivariable logistic regression analysis were used to find potential risk factors of LAE. Results: Ninety-six patients were finally included in this study. The mean age was 54.5 ± 11.7 years and 85 (88.5%) were male. LAE occurred in 15 (15.6%) of 96 patients after TEVAR. Two preoperative factors showed strong associations with LAE according to the multivariable logistic regression analysis: preoperative partial thrombosis of the FL (OR = 10.989 [2.295–48.403]; *p* = 0.002) and the maximum descending aortic diameter (OR = 1.385 [1.100–1.743] per mm increase; *p* = 0.006). Conclusions: Preoperative partial thrombosis of the FL and an increase in the maximum aortic diameter are strongly associated with late aortic expansion. Additional interventions of the FL may help to improve the prognosis of patients with the high risk of late aortic expansion.

## 1. Introduction

Acute type B aortic dissection (TBAD) is a cardiovascular emergent and lethal condition. Open surgery was once used as the first-line treatment option for acute TBAD but the postoperative mortality and complication rates remain high [1]. Since the successful implementation of thoracic endovascular aortic repair (TEVAR), the treatment of acute TBAD has shifted from massively invasive to minimally invasive, with significant reductions in perioperative mortality and complication rates [2,3,4,5,6]. The TEVAR technique can exclude the primary intimal tear (IT) in the proximal descending aorta and improve perfusion of the true lumen (TL) [7,8]. Reopening of the TL and complete regression of the false lumen (FL) are considered ideal for postoperative aortic remodeling [9]. Unfortunately, this situation appears to be a luxury event for most patients with acute TBAD [7]. Patients with a patent FL remain at significant risk for late aortic expansion (LAE) [3,9,10,11]. In patients with a patent FL, it is important to maintain a stable state of the FL and to control the LAE in order to improve the prognosis and survival time of the patients as much as possible. The Society for Vascular Surgery (SVS) and Society of Thoracic Surgeons (STS) reported a worrying set of statistics, showing that more than 60% of patients with acute TBAD, regardless of the initial treatment modality, will develop an aneurysmal growth during the next 5 years [12]. The early identification and management of potential risk factors for LAE is, therefore, particularly important.

Although previous studies have found some possible factors associated with LAE, most of them have focused on the postoperative features while ignoring the preoperative features. The purpose of this study was to investigate whether preoperative features can predict postoperative LAE.

## 2. Materials and Methods

### 2.1. Study Cohort

We retrospectively reviewed 96 acute TBAD patients who presented to the First Affiliated Hospital of Nanjing Medical University from January 2018 to December 2020. Inclusion criteria: (1) patients with acute TBAD; (2) TEVAR technique performed; (3) complete follow-up data. Exclusion criteria: (1) penetrating aortic ulcer; (2) aortic intermural hematoma; (3) aortic aneurysm; (4) previous aortic surgery; (5) Marfan syndrome and other connective tissue diseases; (6) complete regression of the FL. The demographics, clinical characteristics, clinical outcomes, and TEVAR details of 96 patients were collected from the clinical database.

This study was approved by the Institutional Review Board of the First Affiliated Hospital of Nanjing Medical University. The requirement for written informed consent was waived due to the retrospective nature of this investigation.

### 2.2. Imaging Data

**Figure 1:**
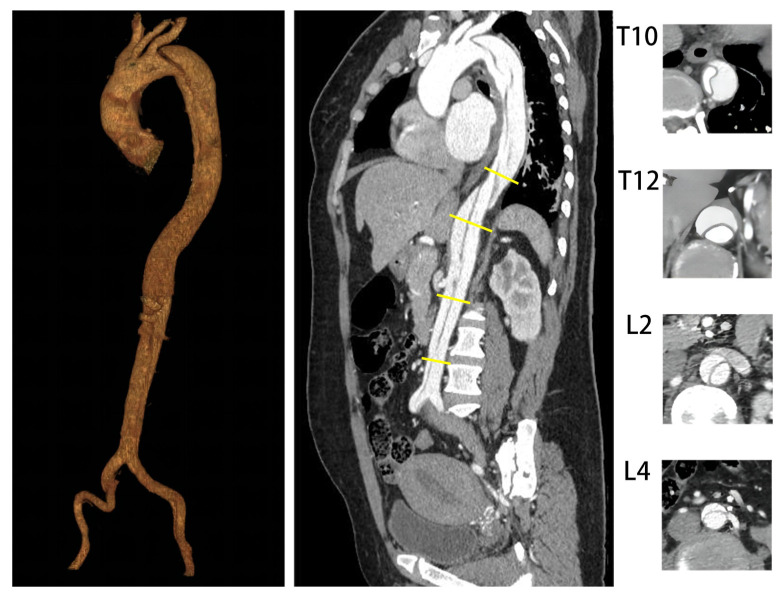
Measurement at four level.

**Figure 2:**
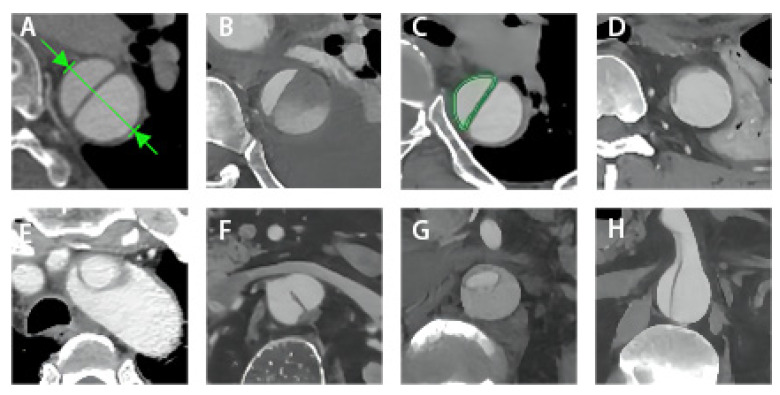
Observation of eight morphologic features: (**A**) measurement of diameter; (**B**) partial thrombosis of the FL; (**C**) TL; (**D**) TL compression; (**E**) dissection extends to the aortic arch; (**F**) untreated tear; (**G**) annular tear; (**H**) dissection extends to abdominal branches. FL, false lumen; TL, true lumen.

All imaging data were obtained through the clinical image database and all baseline and follow-up aortic computed tomography angiography (CTA) scans were analyzed using dedicated 3D image post-processing software (EndoSize station, Version 3.1.67, Therenva SAS, Rennes, France). The follow-up scans were obtained after 1 and 6 months of follow-up and yearly thereafter. The most recent CTA scan was used for the analysis if multiple imaging studies were performed during follow-up. Measurements were obtained at the same levels perpendicular to the line of the intimal flap in four selected aortic segments: T10, T12, L2, and L4 (Figure 1). LAE was defined as an annual aortic growth rate > 5 mm/year after TEVAR [12]. The annual aortic growth rate was calculated at four levels by dividing the difference in maximum aortic diameter between the initial and final measurements by the time interval between the imaging examinations in years. Preoperative partial thrombosis of the FL was defined as the concurrent presence of both flow and thrombus. TL compression was defined as a ratio of the area of the TL to the area of the total aortic lumen of less than 0.3 (Figure 2).

### 2.3. TEVAR Procedures

All 96 patients were under strict electrocardiographic monitoring and optimal medical therapy in hospital. After initial medical management, a surgical plan was made according to the preoperative CTA, and then the TEVAR procedure was performed in a hybrid unit. We selected devices with <10% oversizing based on the diameters of the proximal and distal landing zones of the aorta. Closure or revascularization of the supra-aortic branches was induced as needed to obtain an adequate (>15 mm) proximal landing zone. The access route was transfemoral and achieved using a percutaneous or cutdown technique, and if the supra-aortic branches needed to be reconstructed, the left brachial artery or neck cutdown technique was possibly required. A 0.035-inch guide wire and 5-Fr pigtail catheter were placed into the root of the ascending aorta (AA) via a 5-Fr sheath preplaced into the common femoral artery, and aortic angiography was performed to confirm whether the catheter was placed in the TL. Meanwhile, the diameters of the landing zones and the position of the primary IT were measured again. A Lunderquist Extra Stiff Wire Guide was exchanged in the AA for better support during stent deployment. The endograft delivery system was advanced over the Lunderquist Extra Stiff Wire Guide under fluoroscopy and placed in the desired position. Aortic angiography was performed again to demonstrate the closure of the primary IT and the blood flow of the aortic branches. The endograft delivery system was then retrieved carefully.

### 2.4. Statistical Analysis

Categorical variables are presented as frequency and percentage (%) values and the χ^2^ test or Fisher’s exact test were used as appropriate. Continuous variable data are presented as the mean ± standard deviation or median (interquartile range (IQR); Q25, Q75) and a *t*-test or Mann–Whitney U test was used as appropriate. All variables with *p* values < 0.20 in the univariate analysis or theoretically or previously related to aortic expansion were included in the multivariate model. The data were analyzed using SPSS 26.0 software (IBM Corp., Armonk, NY, USA) and the differences were considered significant at *p* < 0.05.

## 3. Results

### 3.1. Patient Characteristics

We identified a total of 96 patients. The mean age was 54.5 ± 11.7 years and 85 (88.5%) were male. Fifty-three patients (55.2%) presented with chest pain at the onset of symptoms. There was no significant difference between the expanded group and non-expanded group in terms of sex, age, medical history, and underlying disease (*p* > 0.05). The clinical demographics are shown in Table 1. Hypertension was found in 78 patients (81.25%), which included 10 patients (66.7%) in the expanded group and 68 (84.0%) in the non-expanded group (*p* = 0.115). TL compression occurred in 74 patients (77.1%), which included 14 (93.3%) in the expanded group and 60 (74.1%) in the non-expanded group (*p* = 0.103). Preoperative partial thrombosis of the FL was present in 16 patients (16.7%), which included 9 patients (56.1%) in the expanded group (*p* < 0.001). The maximum descending aortic diameter was 41.3 ± 4.5 mm in the expanded group and 37.5 ± 3.0 mm in the non-expanded group (*p* < 0.001). The imaging features are shown in Table 2.

### 3.2. Operative Details

All procedures were completed successfully. The average time from onset to TEVAR was 12.8 ± 6.9 days in the expanded group and 12.4 ± 6.6 days in the non-expanded group (*p* = 0.823). Six patients underwent TEVAR within 24 h of onset. A total of 114 aortic stents were placed in 96 patients, including 6 Valiant stent grafts (Medtronic, Inc, Minneapolis, MN, United States), 17 GORE C-TAG stent grafts (W.L. Gore and Associates, Newark, DE, United States), and 91 Ankura stent grafts (Lifetech Scientific Co., Ltd., Shenzhen, China), with a diameter range of 28 mm to 45 mm and a length range of 80 mm to 200 mm. Eighteen patients (18.8%) received two stents. For the management of the supra-aortic branches, a chimney stent of the LSA was performed in 15 patients, vitro fenestration of the LSA in 18 patients, in situ fenestration of the LSA in 15 patients, in situ fenestration of the LSA and left common carotid artery in 6 patients, and in situ fenestration for all 3 supra-aortic branches in 4 patients.

### 3.3. Postoperative Complications

**Figure 3:**
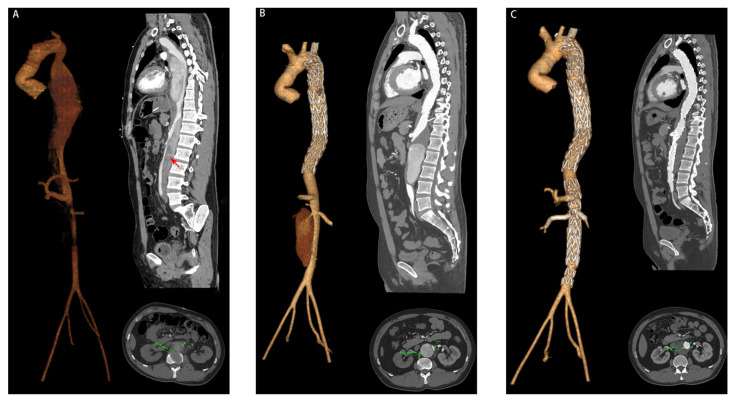
A 43-year-old patient with late aortic expansion at 6 months after TEVAR: (**A**) aortic CTA at onset of symptoms, where the red arrow indicates partial thrombosis of the FL; (**B**) aortic CTA at 6 months after TEVAR and a 12.8 mm increase in aortic diameter compared to the preoperative state; (**C**) aortic CTA at one month after reoperation and complete thrombosis of the FL.

The mean follow-up time was 35.6 ± 11.9 months (range 18–54 months). LAE occurred in 15 patients (15.6%), including seven patients classed in level T10, seven patients in level T12, three patients in level L2, and one patient in level L4 (including two patients classed in both levels T10 and T12 and one patient classed in both levels L2 and L4) (Table 3). Six (40.0%) of the 15 patients with LAE underwent reoperation, including one at 5 months, two at 6 months, one at 8 months, one at 18 months, and one at 20 months, postoperatively (Figure 3). One patient (1.0%) underwent the Bentall procedure for ascending aortic dissection at 16 months after TEVAR. Two patients (2.0%) had postoperative spinal cord injury, and fortunately both recovered; one patient (1.0%) had gastrointestinal bleeding 6 days after TEVAR; one patient (1.0%) underwent an incision of the right lower limb due to osteofascial compartment syndrome; seven patients (7.3%) received temporary hemodialysis treatment after TEVAR, including 4 patients with chronic kidney disease and 3 patients with acute kidney injury (AKI). No death was observed during follow-up.

### 3.4. Univariable and Multivariable Logistic Regression Analysis of the Risk Factors of LAE

The univariable analysis showed that hypertension (*p* = 0.115), TL compression (*p* = 0.103), preoperative partial thrombosis of the FL (*p* < 0.001), and the maximum descending aortic diameter (*p* < 0.001) may relate to LAE. The results of the univariable analysis of the predictors of LAE are given in Table 1 and Table 2. The multivariable logistic regression analysis showed that preoperative partial thrombosis of the FL (OR = 10.989 [2.295–48.403]; *p* = 0.002) and the maximum descending aortic diameter (OR = 1.385 [1.100–1.743] per mm increase; *p* = 0.006) were significantly related to LAE (Table 4).

## 4. Discussion

We studied a relatively homogenous group (with a patent FL at the latest CTA follow-up) in order to minimize the impact of confounding factors. In our cohort of patients with TBAD, the incidence of LAE was 15.6%, and six patients underwent reoperation. Preoperative partial thrombosis of the FL was present in 16 patients (16.7%) and was the strongest independent predictor of LAE that we identified; the risk of LAE in these patients was increased by a factor of 12.470 in comparison with the other patients. Additionally, an increase in the maximum descending aortic diameter was also associated with LAE.

As a revolutionary technique in the treatment of aortic disease in recent decades, TEVAR has been widely used in patients with acute TBAD and has achieved excellent clinical results and prognoses, but there are still a considerable number of patients at risk of secondary dissection progression, especially for patients with a patent FL [2,7,9]. Distal aortic expansion is still a major problem for these patients. Compared with the patients with FL regression, the prognosis of patients with a patent FL was significantly worse, and the risk of LAE increased significantly. Various studies have demonstrated that the characteristics of patients and the anatomy of the aorta determine the growth status after TEVAR. Some studies have investigated the risk factors of aortic expansion. However, most of the previous studies that reported aortic expansion were only focused on the postoperative FL status [13,14,15].

Previous studies have found some risk factors that may lead to late aortic expansion. Tolenaar et al. [16] reported that patients aged <60 years had a significantly higher growth rate (4.2 ± 7.51 mm) than patients aged >60 years (1.8 ± 4.07 mm). A possible explanation for this observation is that the structure of the aortic wall degenerates over time and becomes less elastic due to the increased occurrence of atherosclerosis [17,18]. Genetic connective tissue disease has also been confirmed to be associated with aortic expansion, especially Marfan syndrome [19,20]. FBN1 mutation leads to poor structural and functional integrity of the normal aortic wall. The changes in the walls of the elastic arteries occur primarily in the medial layer and are associated with less distensibility and increased stiffness, leading to consequent weakening and expansion. Distal reentry also has been widely studied [21,22]. Zhang et al. [11] reported that with a closer distance of the first reentry to the LSA, more tears in the thoracic descending aorta and a larger area of reentry appeared to be the key risk factors of late aortic expansion.

Some studies reported that postoperative partial thrombosis of the FL during follow-up was related to LAE. Tsai et al. [23] found that partial thrombosis of the FL was associated with a faster aortic growth rate and a higher rate of reoperation through the study of 67 patients. In a meta-analysis of previous studies, Li et al. [24] found that patients with partial thrombosis of the FL had more complex flow conditions within the FL compared to complete patency, which was more likely to cause turbulence or eddy flow, resulting in increased shear stress in the aortic wall and finally leading to aortic expansion. Burris et al. [25] suggested that an imbalance of blood flow between the inflow and outflow of the FL led to increased pressure in the FL. Their study differed from ours in that they focused on the postoperative FL status, whereas we focused on the preoperative status.

**Figure 4:**
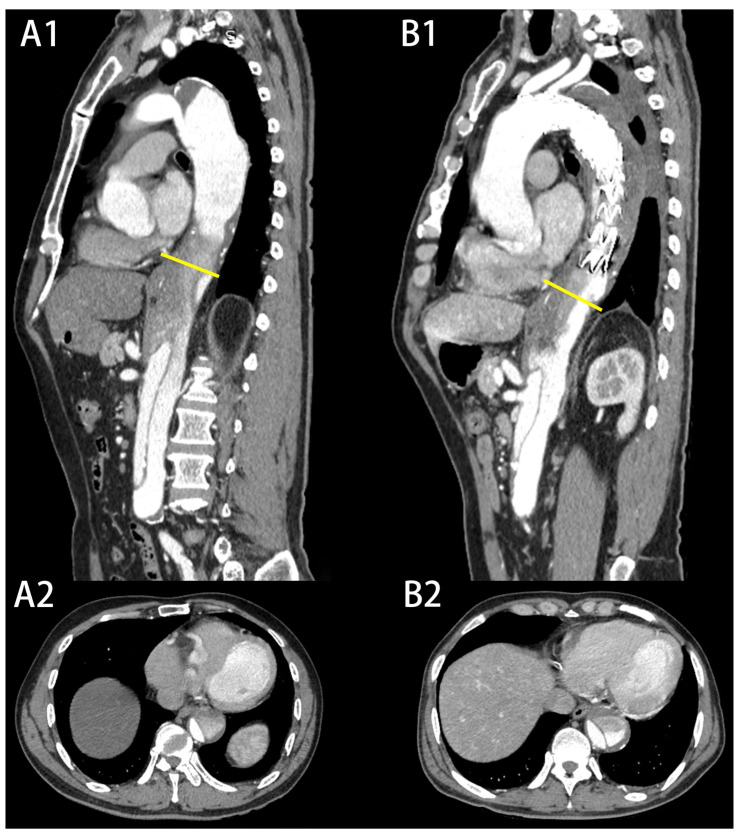
The state of the FL before and after TEVAR: (**A1**,**A2**) a patient with preoperative partial thrombosis of the FL, with no significant change in the state of the FL after TEVAR (**B1**,**B2**).

In this study, we demonstrated that preoperative partial thrombosis of the FL was related to LAE during the follow-up period. However, some researchers believe that this feature may change over time, especially before and after TEVAR, and dynamic follow-up may be more beneficial [26]. In our study, sixteen patients presented preoperative partial thrombosis of the FL and 13 patients remained in the same state after TEVAR (Figure 4). Wojciechowski et al. [2] found that TEVAR is a safe and effective method for TBAD but it protects only the covered segment of the thoracic aorta. This may be the reason why the state of the FL does not change. Meanwhile, we believe that the risk stratification model based on dynamic follow-up or postoperative characteristics is more suitable for patients who receive medical treatment, because these patients undergo surgery at the time that aortic expansion appears. Meanwhile, for patients who are ready for their first TEVAR, an immediate evaluation can enable them to benefit from decision-making, especially for patients at high risk of LAE. Previous studies have explained the causes of aortic expansion caused by partial thrombosis of the FL. First, formation of a thrombus blocks the distal IT and decreases the outflow of the FL, leading to increases in pressure in the FL [24,27]. Second, the formation of the thrombus activates the coagulation system, while hypoxia in the arterial wall near the thrombus leads to increased local inflammation, neovascularization, and local weakening of the vessel wall [28]. Third, FL thrombus formation is the consequence of slow, stagnating blood flow [29] due to poor FL drainage or outflow, which is associated with increased diastolic pressure and in turn faster aneurysmal degeneration [26,30]. The real reasons for aortic expansion are still unknown, and it may be the result of many factors. In our study, we observed that a thrombus usually first forms in the wall of the aorta far from the IT and gradually progresses to the center. A blind end is gradually formed, and the inflow and outflow of the FL are not balanced, which leads to late aortic expansion.

However, not all studies have come to this consistent conclusion. Sueyoshi et al. [31] reported that partial thrombosis of the FL is not a significant risk factor for enlargement, although the aortic growth rate in the partial thrombosis of FL group was higher than the complete thrombosis group (4.0 ± 4.3 mm/year vs. −0.2 ± 0.6 mm/year). The same conclusion was reached in another study by Larsen et al. [23], wherein the median aortic growth rates for groups with complete thrombosis of the FL, partial thrombosis of the FL, and a patent FL were 0.3 (−0.5 to 2.4), 2.1 (−0.1 to 4.9), and 1.8 (0.8–4.0) mm/year. Although their interpretation of the conclusion was different from ours, they were still consistent regarding the issue of the aortic growth rate. As for the different conclusions, we think the diverse formations of partial thrombosis should be considered, such as whether the formation is a sac-type or a non-sac type, whether communication with the main arterial branches exists or not, the proportion of thrombosed lumen to false lumen, or the volume of the thrombosis and location, although this information remains speculative [15,32]. On the other hand, there are many confounding factors that affect the final conclusion, which may also lead to the inconsistency of the final conclusion.

The results of our study also suggest that the factors associated with LAE in the radiologic findings include increases in maximum aortic diameter, which have been frequently reported in many previous studies. Despite these differences in the technique of measurement of the aortic diameter, there was a good agreement among the studies about the predictive value for late aortic expansion and mortality. The cutoff was set at 40 mm in most studies. Hata et al. [33] suggested that with the increase in the aortic diameter, patients face a greater risk of aortic expansion, and a maximum aortic diameter exceeding 40 mm at the time of TBAD onset was the only significant predictor of elective surgery. Meanwhile, there long-term outcomes showing a 10-year freedom from surgery rate of 76.0% with aggressive hypotensive therapy and close monitoring are satisfactory. It is worth noting that Miller et al. [34] performed a subgroup analysis based on height, finding that the 40-mm cutoff was associated with a complicated course in patients shorter than 180 cm, but was a nonsignificant predictor for patients taller than 180 cm. Higashigaito et al. [26] suggested that morphologic risk factors need to be interpreted in the context of the entire preceding morphologic evolution. Patients with a larger aortic diameter tended to have a larger FL and a smaller TL in our study. This makes it difficult for the TL to reopen after TEVAR while the FL continues to grow. Therefore, it is controversial whether more aggressive treatment should be taken for patients with a large initial aortic diameter.

Previous studies have shown some risk factors leading to aortic expansion, and most scholars still recommend hypotensive therapy and close monitoring at follow-up rather than other proactive treatments. In recent years, with the development and progress of technology, FL interventions have been used in clinics to actively induce FL thrombosis and improve the prognosis of patients [35,36,37]. By actively placing a specific device, such as coils or atrial septal occluders, in the false lumen, this can promote remodeling and thrombosis of the FL and improve the prognosis of the patients, but the indications and long-term prognosis of FL interventions remain to be further studied. At present, there is no medical instrument that is specially used for FL interventions, and the most widely used devices are coils. New special equipment for FL interventions needs to be developed. Acute TBAD after TEVAR should also be treated as a chronic disease because of the long-term risk of LAE [12]. Attention should be paid to whether the scope of false lumen thrombosis has changed during follow-up. Beta-blockers and angiotensin-1 antagonists have been seen to be associated with reduced aneurysmal degeneration of the dissected aorta and promotion of complete false lumen thrombosis. Identifying high-risk patients at an early stage deserves consideration by all clinicians.

## 5. Limitations

Our study has several limitations. First, the retrospective design and the relatively small population size may have influenced the final results. Second, this was a single-center study, and it might not be applicable to explain the results of other centers. Third, the process of aortic expansion after TEVAR is influenced by various factors, and we were unable to collect all possible factors, despite our efforts to do so.

## 6. Conclusions

In conclusion, preoperative partial thrombosis of the FL and an increase in the maximum descending aortic diameter seem to be associated with late aortic expansion after TEVAR. Identifying the risk factors before TEVAR and reconsidering the TEVAR plan may benefit the patients more.

## Figures and Tables

**Table 1:** Characteristics of 96 patients stratified according to whether late aortic expansion occurred.

Characteristic	Expanded Group (*n* = 15)	Non-Expanded Group (*n* = 81)	*p* Value
Sex			
Male, *n* (%)	14 (93.3)	71 (87.7)	0.526
Age (year), mean ± SD	53.1 ± 10.6	54.8 ± 11.9	0.598
Smoking, *n* (%)	5 (33.3)	25 (30.9)	0.850
Hyperlipidemia, *n* (%)	4 (26.7)	16 (19.8)	0.545
BMI (kg/m^2^), mean ± SD	27.1 ± 4.4	25.9 ± 4.3	0.336
Hypertension, *n* (%)	10 (66.7)	68 (84.0)	0.115
Diabetes, *n* (%)	2 (13.3)	9 (11.1)	0.804
CAD, *n* (%)	1 (6.7)	9 (11.1)	0.605
Atherosclerosis, *n* (%)	7 (46.7)	32 (39.5)	0.604
CVD, *n* (%)	0 (0.0)	4 (4.9)	0.379
VHD, *n* (%)	1 (6.7)	4 (4.9)	0.782
CKD, *n* (%)	1 (6.7)	7 (8.6)	0.799
Symptoms			0.488
Chest pain, *n* (%)	9 (60.0)	44 (54.3)	
Abdominal pain, *n* (%)	3 (20.0)	9 (11.1)	
Back pain, *n* (%)	1 (6.7)	18 (22.2)	
Asymptomatic, *n* (%)	2 (13.3)	10 (12.3)	
Chronicity classification of AD			0.521
Hyperacute, *n* (%)	0 (0.0)	6 (7.4)	
Acute, *n* (%)	11 (73.3)	58 (71.6)	
Subacute, *n* (%)	4 (26.7)	17 (21.0)	
ASA Classification			0.407
I, *n* (%)	0 (0.0)	8 (9.9)	
II, *n* (%)	2 (13.3)	10 (12.3)	
III, *n* (%)	9 (60.0)	53 (65.4)	
IV, *n* (%)	4 (26.7)	9 (11.1)	
V, *n* (%)	0 (0.0)	1 (1.2)	
Onset to TEVAR (days), mean ± SD	12.8 ± 6.9	12.4 ± 6.6	0.823

Data are presented as the mean ± standard deviation (SD) or *n* (%). BMI, body mass index; CAD, coronary artery disease; CVD, cerebrovascular diseases; VHD, valvular heart disease; CKD, chronic kidney disease; AD, aortic dissection; ASA, American Society of Anesthesiologists; TEVAR, thoracic endovascular aortic repair.

**Table 2:** Imaging features of 96 patients.

Characteristic	Expanded Group (*n* = 15)	Non-Expanded Group (*n* = 81)	*p* Value
Annular tear, *n* (%)	2 (13.3)	6 (7.4)	0.446
Dissection extends to aortic arch, *n* (%)	2 (13.3)	13 (16.0)	0.790
Untreated tears, mean ± SD	1.87 ± 0.99	1.78 ± 0.85	0.748
Diameter of stents (mm), range	32.0 (32.0, 34.0)	32.0 (31.5, 36.0)	0.761
Length of stents (mm), median (IQR)	192.0 (189.0, 197.0)	194.0 (189.5, 200)	0.223
Dissection extends to abdominal branches			
Coeliac trunk, *n* (%)	6 (40.0)	43 (53.1)	0.352
SMA, *n* (%)	1 (6.7)	11 (13.6)	0.457
RA, *n* (%)	7 (46.7)	38 (46.9)	0.986
Dissection extends to CIA, *n* (%)	12 (80.0)	60 (74.1)	0.626
TL compression, *n* (%)	14 (93.3)	60 (74.1)	0.103
Partial thrombosis of FL, *n* (%)	9 (60.0)	7 (8.6)	<0.001
Maximum descending aortic diameter (mm), mean ± SD	41.3 ± 4.5	37.5 ± 3.0	<0.001

Data are presented as the mean ± standard deviation (SD), median (IQR), or *n* (%). FL, false lumen; SMA, superior mesenteric artery; RA, renal artery; CIA: common iliac artery; TL, true lumen; FL, false lumen.

**Table 3:** Changes in aortic diameter at four levels after TEVAR.

Level of Aorta	Group	Before TEVAR	Follow-Up
		Mean ± SD (mm)	Mean ± SD (mm)
T10	Expanded group	40.1 ± 4.2	44.0 ± 4.0
	Non-expanded group	36.3 ± 3.8	36.1 ± 4.4
T12	Expanded group	35.8 ± 3.7	40.6 ± 5.3
	Non-expanded group	34.4 ± 2.8	35.1 ± 3.2
L2	Expanded group	30.0 ± 3.3	32.5 ± 2.7
	Non-expanded group	28.8 ± 2.4	29.3 ± 2.4
L4	Expanded group	27.5 ± 2.9	28.7 ± 3.0
	Non-expanded group	24.2 ± 2.5	24.7 ± 2.7

Data are presented as the mean ± standard deviation (SD). TEVAR, thoracic endovascular aortic repair.

**Table 4:** The multivariable analysis of the risk factors of late aortic expansion.

Characteristic	OR	95%CI	*p* Value
Hypertension	0.495	0.102–2.395	0.382
TL compression	6.301	0.340–116.920	0.217
Maximum descending aortic diameter (mm)	1.385	1.100–1.743	0.006
Partial thrombosis of FL	10.989	2.295–48.403	0.002

OR: odds ratio; CI: confidence interval; TL, true lumen; FL, false lumen.

## Data Availability

The study data will be made available upon request to the corresponding author.
